# Quality of life and fear of COVID-19 in 2600 baccalaureate nursing students at five universities: a cross-sectional study

**DOI:** 10.1186/s12955-021-01837-2

**Published:** 2021-08-19

**Authors:** E. G. Beisland, K. H. Gjeilo, J. R. Andersen, O. Bratås, B. Bø, K. Haraldstad, I. H. H. Hjelmeland, M. M. Iversen, B. Løyland, T. M. Norekvål, K. Riiser, G. Rohde, K. H. Urstad, I. Utne, T. N. Flølo, the LIVSFORSK network

**Affiliations:** 1 Department of Health and Caring Sciences, Western Norway University of Applied Sciences, Inndalsveien 28, 5063 Kronstad, Bergen, Norway; 2 Department of Public Health and Nursing, Norwegian University of Science and Technology, NTNU, 7491 Trondheim, Norway; 3 Department of Quality and Health Technology, University of Stavanger, Kjell Arholms gate 41, 4021 Stavanger, Norway; 4 Department of Health and Nursing Sciences, University of Agder, P.O Box 422, 4604 Kristiansand, Norway; 5 Department of Nursing and Health Promotion, Oslo Metropolitan University, St. Olavs plass, P.O. Box 4, 0130 Oslo, Norway; 6 Department of Physiotherapy, Oslo Metropolitan University, St. Olavs plass, P.O. Box 4, 0130 Oslo, Norway; 7 Department of Surgery, Voss Hospital, Haukeland University Hospital/The Western Norway Health Region Authority, Sjukehusvegen 16, 5704 Voss, Norway; 8 Department of Cardiology and Department of Cardiothoracic Surgery, St. Olav Hospital, Trondheim University Hospital, Postbox 3250, 7006 Torgarden, Trondheim, Norway; 9 Department of Heart Disease, Haukeland University Hospital, Jonas Lies vei 65, 5021 Bergen, Norway; 10 Department of Clinical Research, SSHF, P.O. Box 416, 4604 Kristiansand, Norway; 11 Førde Hospital Trust, Post Office Box 1000, 6807 Førde, Norway

**Keywords:** Fear of COVID-19, Quality of life, General health, Psychological distress, Nursing students

## Abstract

**Background:**

The COVID-19 pandemic has caused significant disruptions in the implementation of programs across educational institutions. Nursing students, being both young adults and by practical training, part of the health care system, may be particularly vulnerable during the COVID-19 pandemic. The purpose of this study was to explore the associations between self-reported fear of COVID-19, general health, psychological distress and overall quality of life (QoL) in a sample of Norwegian baccalaureate nursing students compared to reference data.

**Methods:**

The survey targeted baccalaureate nursing students from five universities in February 2021. An electronic questionnaire consisted of the Fear of COVID-19 Scale (FCV-19S), the Hopkins Symptom Checklist 5 (SCL-5), one general health and one overall QoL question. The respondents’ mean scores were compared to reference data. Hierarchical regression analyses were conducted, and effect sizes (Cohen’s d) were evaluated.

**Results:**

In total, 2605 out of 6088 (43%) students responded. Their FCV-19S scores (mean 2.45, CI 2.42, 2.48) were significantly higher than those of the reference population (mean 1.8, *P* < 0.001). Nursing students scores showed significantly lower general health (mean 3.50 ± 0.93 SD, population mean = 3.57, Cohen’s d = 0.07), higher levels of psychological distress (mean 2.68 ± 1.03 SD, population mean = 2.12, Cohen’s d = 0.55) and lower overall QoL (mean 5.50 ± 2.16 SD, population mean = 8.00, Cohen’s d = 1.16) compared to pre-pandemic reference data. FCV-19S scores were significantly associated with levels of general health (Cohen’s d = 0.26), psychological distress (Cohen’s d = 0.76) and overall QoL (Cohen’s d = 0.18).

**Conclusions:**

Baccalaureate nursing students reported worse outcomes during the Covid-19 pandemic on general health, psychological distress and overall QoL compared to the reference population. Level of fear of Covid-19, however, accounted for few of these differences. Other factors related to the pandemic may have reduced nursing students’ overall QoL.

**Supplementary Information:**

The online version contains supplementary material available at 10.1186/s12955-021-01837-2.

## Introduction

The coronavirus disease 2019 (COVID-19) pandemic has reached unforeseen dimensions and will have far-reaching implications for quality of life (QoL) into the future [1]. Mental health and QoL have been affected globally, in the general population [1–3], patients and other vulnerable groups [1, 4] as well as in health care workers [5–7]. The World Health Organization (WHO) defines QoL as ‘an individual’s perception of their position in life, in the context of the culture in which they live and in relation to their goals, expectations, standards and concerns’ [8]. Mental health may be hampered by concern about one’s personal health or by worries about family members and friends, and in the case of health care providers, concern for patients. Furthermore, mental health may be hampered by stress, frustration or annoyance about being restricted as part of precautionary measures, such as prolonged lockdown periods and social distancing and school closures or the use of quarantine [9–11].

Mental health may be challenged in a dual manner during a pandemic among health care providers, as they are exposed to the risk of infection both professionally and in their private lives [12, 13]. A recent systematic review and meta-analysis reported pooled prevalence estimates of stress, anxiety, depression and sleep disturbances among nurses during the COVID-19 outbreak. More than one third of nurses reported higher scores on these measures than those reported during the previous Middle East Respiratory Syndrome (MERS) and Severe Acute Respiratory Syndrome epidemics [5].

Measures of anxiety and depression using different instruments are generally more prevalent in college students than in the general population as such [9, 14, 15]. In a 2018 nationwide survey of Norwegian students in a higher education setting, their health and psychological distress were studied [16]. A worrisome increase in self-reported psychological distress over recent years was found, as assessed by the Hopkins Symptom Checklist (SCL-25). The scores were markedly higher for women than for men at all time-points [16]. A Norwegian survey of QoL and psychological distress during the COVID-19 pandemic found a general decline in measures of life-satisfaction and mental health [17]. Young adults and students seemed to be more severely affected by the pandemic, for instance, with regard to feelings of loneliness.

The pandemic has caused significant disruptions in the implementation of programs across educational institutions. Nursing students, being both young adults and by practical training, part of the health care system, may be particularly vulnerable during the COVID-19 pandemic [18–20]. In a Turkish study, nursing students reported increased levels of stress related to the COVID-19 pandemic [21]. A Mexican study found that nursing students and recent graduates had high levels of stress and fear, in addition to a low level of knowledge. The presence of high stress and low knowledge predicted fear regarding COVID-19 [22].

Norwegian baccalaureate nursing students have been affected by different degrees of restrictions depending on local and temporal variations in the incidence of infection. To maintain academic progress during the pandemic’s lockdown periods, educational sessions have largely been converted to digital teaching. Practical training, which is normally 50% of the three-year baccalaureate nursing programme (a total of 180 European Credit Transfer System points), has proceeded with modifications. Follow-up of students during clinical practice has been implemented mainly via digital platforms. In some parts of the health care system, clinical practice has been shortened or altered to practice in simulation arenas at university campuses.

Validated and reliable tools for the assessment of an individual’s fear have emerged during the COVID-19 pandemic [23–25]. Ahorsu et al. [26] developed the Fear of COVID-19 Scale (FCV-19S) with an Iranian population, which has been validated in a Norwegian sample of the general population [27]. To our knowledge, the scale has, so far, been used with a small sample of baccalaureate nursing students from the Philippines, to investigate the associations between fear of COVID-19 and the intention to quit school [28].

In this study we aimed to explore whether fear of COVID-19 is associated with self-reported general health, psychological distress and overall QoL in a sample of Norwegian baccalaureate nursing students. The use of established instruments to assess these outcomes, allowed us to compare our findings to reference data on students reported prior to the COVID-19 pandemic.

## Methods

### Design and sample

Between 27th January and 28th February, 2021, we invited all full- and part-time baccalaureate nursing students > 18 years of age from five Norwegian universities at ten different campuses (N = 6088) to take part in a web-based cross-sectional survey. The participating universities were Oslo Metropolitan University, Western Norway University of Applied Sciences, the University of Agder, the Norwegian University of Science and Technology and the University of Stavanger.

### Measures

The survey included questions related to students’ demographics, personal health and study situation during the pandemic, specifically developed for the present research by an expert group consisting of clinicians, nursing students, university staff and researchers. Additional measures included four validated instruments for assessing fear of COVID-19, overall QoL, general health and psychological distress.

*Characteristics of the respondents* included age (< 25, 25–29, ≥ 30 years), household status, study site and year of study.

*COVID-19 specific questions related to personal health* were developed for the present study and included the number of times the student was tested for COVID-19 (never, 1, 2, 3 or ≥ 4 times); quarantine history (never, previous, present); feelings of loneliness due to COVID-19 (rated from 1 [strongly disagree] to 5 [strongly agree]); perceived risk for complications of COVID-19 (no, uncertain, yes); history of suspected, possible or confirmed COVID-19 infection; intention to take the vaccine (already taken, yes, undecided, no); and trust in authorities’ and universities’ handling of the pandemic (rated from 1 [strongly disagree] to 5 [strongly agree]).

*COVID-19 specific questions related to education* addressed students’ perceived impact of the different aspects of their education, especially the impacts related to the conduct of clinical training and placements.

*The Fear of COVID-19 Scale (FCV-19S)* [26], which had been adapted and assessed for use with Norwegian samples, was used [27]. Seven items (e.g. ‘I am most afraid of the coronavirus’) are rated on a 5-point scale from 1 (strongly disagree) to 5 (strongly agree), with a total score ranging from 7 to 35. Higher scores represent greater fear of COVID-19. In the present study, the average item score was used; it was calculated by dividing the total score by the number of items.

*The Hopkins Symptom Checklist (SCL-5)* [29] is available as a Norwegian translation [30]. It consists of five items measuring psychological distress (anxiety and depression) that are rated on a five-point scale from 1 (not at all) to 5 (extremely). The average item score was calculated by dividing the total score by the number of items answered [15]. Higher scores represent greater psychological distress.

*General health* was assessed using one item derived from the 36-Item Short-Form Health Survey (SF-36) [31], ‘In general, would you say your health is: excellent, very good, good, fair or poor?’ Responses were rated on a five-point scale ranging from 1 (excellent) to 5 (poor) [32]. Consistent with the SF-36 scoring algorithm, the scale was reversed scored [33]. Thus, higher scores reflect better general health, as perceived by respondents. The item was found to be as valid and reliable as multi-item scales [32].

*Overall quality of life* was rated on an adapted version of the Cantril Ladder, on a scale from 0 (not at all satisfied) to 10 (highly satisfied). A score of 6 or more indicates ‘high life satisfaction’ [34]. The question, ‘All in all, how satisfied are you with your life at this time?’, has been widely used in various populations and in different settings; it is considered a valid and reliable measure of overall QoL [35].

Participants’ results on the FCV-19S, SCL-5 and measures of general health and overall QoL were compared to raw reference data, according to the methodology described by Hjermstad et al. [36]. At present, the FCV-19S data from the general population are unavailable. Thus, to compare the students’ score on the FCV-19S we used scores from an urban adult Norwegian population (n = 1063, 12.1% of the youngest (18–29 years) and 55.3% female responders) [27]. Participants’ results on SCL-5, general health and overall QoL were compared to original data from three reference populations. The mean scores for the reference populations were adjusted for sex and age and compared to our participants’ results using one-sample t-tests as described by Hjermstad et al. [36]. For the SCL-5, the nursing students’ scores were compared to those of Norwegian first-year medical students (n = 169, mean age 22.5 and 75% female responders) [15]. For general health our sample of nursing students was compared to the Norwegian general population reporting on an identical question covered by the SF-36 (2118, mean age 55.7 years, but the response rate of the youngest age group (18–29 years) was only 5% and 54% were female) [31] For overall QoL, reference data was available from the Norwegian Survey on Living Conditions (n = 6179, mean age 48.5 ± 18.5, and 49% female responders) [37].

The questionnaire was piloted with 9 nursing students, and after minor adjustments, a digital pilot study was conducted with 90 physiotherapy students. No adjustments were made after the digital pilot. A brief description of the study and an invitation to the web-based survey was e-mailed to 6088 baccalaureate nursing students’ registered university e-mail addresses and made available on the respective learning portals of their teaching institution. At two universities, additional announcements were made at the students’ common Facebook site. All students received at least two reminders by e-mail.

The front page of the survey contained a detailed description of the study and information about voluntary participation. By completing and submitting the survey, the students consented to participate. All responses were stored automatically in ‘SurveyXact’ (https://www.surveyxact.com). The respondents’ IP addresses were not registered and their answers could not be linked to their identities in any way; thus, their participation was anonymous and ethical approval not required according to Norwegian legislation. The survey was evaluated by the Data Protection Officer at the responsible institution, i.e. Western Norway University of Applied Sciences, with additional approval of each university.

### Statistical analyses

Categorical variables are expressed as percentages and continuous variables as means and standard deviations (SD). The FCV-19S scores were stratified by sample characteristics, using separate one-way analysis of variance (ANOVA) tests. Differences between the sample and reference data were investigated using a one-sample t-test. Reference data, except for FCV-19S scores, were adjusted to reflect the age and gender distributions of the respondents, assuming the proportion of males was similar to that of the general nursing student population (approximately 10%). Cohen’s d was used to calculate the effect sizes of the comparisons of means. Unadjusted and fully adjusted hierarchical regression analyses, with the universities as clusters, were conducted to investigate the associations between the FCV-19S score as the independent variable, and the SCL-5 general health or overall QoL score as the dependent variable in separate models. In the regression analysis we standardised the FCV-19S and the three dependent variables where the mean = 0 and standard deviation = 1 (dependent variables were transformed to z-scores, unstandardised regression coefficients). From the fully adjusted models, the associations between other items from the questionnaire and the SCL-5, general health and overall QoL as dependent variables were assessed and reported separately if they had meaningful effect sizes, as assessed by Cohen’s d. A meaningful Cohen’s d was judged to be a difference ≥ 0.2 SD of the dependent variable per 2 SD changes in the FCV-19S or between respondents representing the lower or higher end of the discrete variables with 2–5 categories [38, 39].

Overall, the effect sizes were interpreted as follows: trivial (< 0.2), small (0.2 to < 0.5), moderate (0.5 to < 0.8) and large (≥ 0.8)[40]. We reported two-tailed P-values and 95% confidence intervals (CI) as continuous indicators of the robustness of the estimates. Survey data were downloaded to Microsoft® Excel®, manually coded, and then transferred to IBM SPSS (Statistics for Windows, Version 27.0. Armonk, NY: IBM Corp) for the statistical analyses.

## Results

In total, 2605 of the 6088 students responded to the survey, yielding a response rate of 43%, differing between the universities from 21 to 50%. Among these, 41% (n = 1077), 31% (n = 801) and 28% (n = 730) were baccalaureate students in their programmes’ first, second and third years, respectively. For the seven items in the FCV-19S, Cronbach’s alpha was 0.87 (ranging from 0.84 to 0.86 if single items were deleted), and for the five items in the SCL-5, Cronbach’s alpha was 0.88 (ranging from 0.84 to 0.87 if single items were deleted).

### Fear of COVID-19 Scale

Sample characteristics and mean FCV-19S scores are presented in Table 1. The mean FCV-19S score of our sample of nursing students was 2.45 ± 0.8, compared to 1.85 in the reference population [27]. This difference was statistically significant (*P* < 0.001) with a moderate effect size (Cohen’s d = 0.75) (Table 2).

**Table 1:** Baccalaureate nursing students’ characteristics and level of fear of COVID-19 (N = 2605)

	Number of respondents	Percent respondents	Mean FCV-19S ± SD^b^	*P* value^a^
University A (n = 1893)				< 0.001
B (n = 1796)	938	36	2.57 ± 0.85	
C (n = 858)	874	34	2.41 ± 0.79	
D (n = 675)	396	15	2.32 ± 0.71	
E (n = 866)	214	8	2.38 ± 0.72	
	184	7	2.44 ± 0.80	
Years in the baccalaureate nursing programme				< 0.001
1	1074	41	2.54 ± 0.82	
2	801	31	2.40 ± 0.81	
3	730	28	2.45 ± 0.80	
Age, years				< 0.001
< 25	1846	71	2.50 ± 0.80	
25–29	377	14	2.37 ± 0.82	
≥ 30	382	15	2.27 ± 0.74	
Living alone				0.663
No	2140	82	2.45 ± 0.80	
Yes	465	18	2.47 ± 0.80	
Number of times tested for COVID-19				0.589
Never	765	29	2.44 ± 0.81	
1	724	28	2.43 ± 0.80	
2	445	17	2.43 ± 0.81	
3	325	12	2.47 ± 0.81	
≥ 4	346	13	2.51 ± 0.77	
History of a positive COVID-19 test				0.077
No	2482	95	2.45 ± 0.80	
Yes	110	5	2.57 ± 0.82	
Quarantine status related to COVID-19				0.145
Never	1302	50	2.42 ± 0.80	
Previous	1253	48	2.48 ±0.80	
Now	150	2	2.56 ± 0.91	
At risk for COVID-19 complications				< 0.001
No	2091	80	2.36 ±0.76	
Uncertain	324	13	2.80 ± 0.82	
Yes	190	7	2.81 ± 0.95	
Trust in the government’s handling of the COVID-19 situation				< 0.001
Strongly disagree/disagree	232	9	2.56 ± 0.97	
Neither disagree nor agree	561	22	2.53 ± 0.82	
Agree	1344	52	2.45 ± 0.79	
Strongly agree	468	18	2.30 ± 0.80	
Trust in the universities’ handling of the COVID-19 situation				< 0.001
Strongly disagree	182	7	2.60 ± 0.96	
Disagree	447	17	2.51 ± 0.85	
Neither disagree nor agree	783	30	2.53 ± 0.80	
Agree	984	38	2.36 ± 0.82	
Strongly agree	213	8	2.29 ± 0.80	
Feeling lonely due to COVID-19				< 0.001
Strongly disagree	165	6	1.94 ± 0.70	
Disagree	380	15	2.18 ± 0.72	
Neither disagree nor agree	446	17	2.29 ± 0.71	
Agree	899	34	2.45 ± 0.74	
Strongly agree	718	28	2.81 ± 0.84	
Engagement in clinical practice during the pandemic				< 0.001
Yes	1591	61	2.41 ± 0.79	
No	1014	39	2.52 ± 0.80	

FCV-19S: Fear of Covid-19 Scale. Higher score on Fear of COVID-19 scale (FCV-19S) (1–5) reflects greater fear of COVID-19. University A: Oslo Metropolitan University, B: Western Norway University of Applied Sciences, C: University of Agder, D: Norwegian University of Sciences and Technology, E: University of Stavanger

^a^Unadjusted *P *values for between-groups differences (one way ANOVA)

^b^Standard deviation

**Table 2:** Self-reported fear of COVID-19, general health, psychological distress and overall quality of life in bachelor nursing students versus reference data

Variables	Sample, mean ± SD	Population, mean^c^	Cohen’s d (95% CI^b^)	*P* value*
FCV-19S^a,g^ (1–5)	2.45 ± 0.80	1.85	0.80 (0.70, 0.79)	< 0.001
General health^d^ (1–5)	3.50 ± 0.93	3.57	− 0.07 (− 0,11, − 0.03)	< 0.001
Psychological distress (SCL-5)^e^ (1–5)	2.68 ± 1.03	2.12	0.55 (0.51, 0.59)	< 0.001
Overall Quality of life^f^ (0–10)	5.50 ± 2.16	8.00	− 1.16 (− 1.21, − 1.11)	< 0.001

^a^One-sample t-test mean sample score and unadjusted norm score FCV-19S (Iversen et al. 2021)

^b^Confidence interval

^c^Adjusted for age and gender

*One sample student’s t-test

^d^In line with the SF-36 scoring algorithm, the item was reversed. Higher score reflects better perceived general health

^e^Higher score on Hopkins Symptom Checklist (SCL-5) reflect more psychological distress

^f^Higher score of overall quality of health reflect better perceived overall quality of life

^g^Higher score of FCV-19S reflect higher level of fear of COVID-19

**Fig. 1:**
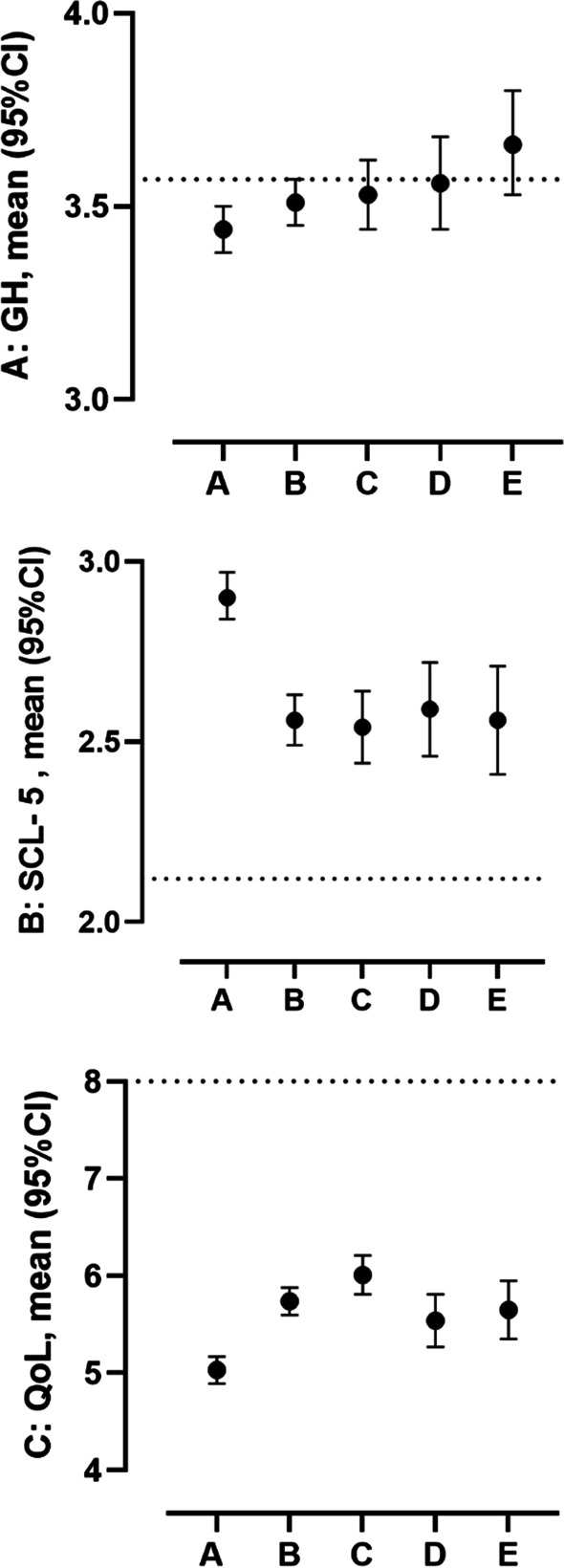
**a** Mean and 95% CI scores of general health (GH) ranging from 1 (worst) to 5 (best). (Originally scored from 1 (excellent) to 5 (poor), but in line with the SF-36 scoring algorithm, the item was reversed so higher scores reflect better perceived general health). **b** Psychological distress (SCL-5) ranging from 1 (best) to 5 (worst) and, **c** Overall quality of life (QoL) ranging from 0 (worst) to 10 (best) stratified by university clusters (n = 5). University A: Oslo Metropolitan University, B: Western Norway University of Applied Sciences, C: University of Agder, D: Norwegian University of Sciences and Technology, E: University of Stavanger. Dashed lines in the respective figures represent the age and gender adjusted reference data scores

Compared to the gender- and age-adjusted reference data, the nursing students’ data showed significantly worse scores on general health, psychological distress and overall QoL (Table 2, Fig. 1). In terms of effect size, the difference was trivial for general health, moderate for psychological distress (SCL-5) and large for overall QoL (Additional file 1).

Eight out of twelve background variables were significantly associated with fear of COVID-19: year in nursing school, younger age, being at risk for COVID-19 complications, lower trust in the government’s and universities’ handling of the pandemic, feeling lonely due to COVID-19 and not being in clinical practice during the pandemic. Fear of COVID-19 differed significantly among the universities.

### General health

Fear of COVID-19 was significantly associated with general health in the adjusted analysis, with a small effect size of − 0.26 SD difference in general health associated with a 2 SD increase in the FCV-19S score (Table 3).

**Table 3:** Hierarchical regression analysis showing associations between fear of COVID-19 (independent variable) and general health, psychological distress and quality of life (dependent variables)

Models	Standardized coefficient (95% CI^a^)	*P* value
*General health*		
FCV-19S: Unadjusted estimate	− 0.23 (− 0.27, − 0.20)	< 0.001
FCV-19S: Adjusted estimate	− 0.13 (− 0.17, − 0.09)	< 0.001
*Psychological distress (SCL-5)*		
FCV-19S: Unadjusted estimate	0.51 (0.47, 0.54)	< 0.001
FCV-19S: Adjusted estimate	0.38 (0.34, 0.41)	< 0.001
*Overall quality of life*		
FCV-19S: Unadjusted estimate	− 0.27 (− 0.30, − 0.23)	< 0.001
FCV-19S: Adjusted estimate	− 0.09 (− 0.13, − 0.06)	< 0.001

FCV-19S: Fear of COVID-19 scale. Psychological distress as measured by the SCL-5: The five item Hopkins symptom checklist. Cluster effects are accounted for in the unadjusted analysis, while the adjusted analyses are adjusted for years in nursing school, age, living alone or not, times tested for COVID-19, history of a positive COVID-19 test, quarantine status related to COVID-19, at risk for COVID-19 complications or not, level of trust in governmental handling of the COVID-19 situation, level of trust in universities’ handling of the COVID-19 situation, feeling of loneliness due to COVID-19 and whether or not the student has engaged in clinical practice during the pandemic. All dependent variables have been transformed to z-scores. Unstandardized regression coefficients

^a^*CI* confidence interval

Other variables from the fully adjusted analysis that were significantly associated with general health and had a meaningful effect size were: being at risk for COVID-19 complications (yes versus no [reference]: standardised score = − 0.77 [95% CI − 0.91, − 0.63]), feelings of loneliness (highest level versus lowest [reference]: standardised score = − 0.48 [95% CI − 0.64, − 0.31]) and level of trust in the government (lowest versus highest [reference]: standardised score = − 0.37 [95% CI − 0.52, − 0.22]) (Additional file 1: Table 1).

### Psychological distress

Fear of COVID-19 was significantly associated with psychological distress in the adjusted analysis, with a moderate effect size of 0.76 SD difference in the SCL-5 per 2 SD increase in the FCV-19S score (Table 3).

Other variables from the fully adjusted model that were significantly associated with psychological distress and had a meaningful effect size were: age (< 25 years old versus ≥ 30 years [reference]: standardised score = 0.24 [95% CI − 0.15, 0.33]), feelings of loneliness (highest level versus lowest [reference]: standardised score = 1.01 [95% CI 0,87, 1.15]) and level of trust in the government (lowest versus highest [reference]: standardised score = 0.28[95% CI 0.15, 0.41] (Additional file 1: Table 2).

### Overall quality of life

Fear of COVID-19 was significantly associated with overall QoL in the adjusted analysis, but with a trivial effect size of − 0.18 SD difference in overall QoL per 2 SD increase in the FCV-19S score (Table 3).

Other variables significantly associated with overall QoL, and with a meaningful effect size, were feelings of loneliness (highest level versus lowest [reference]: standardised score = − 1.38 [95% CI − 0.52, − 1.23]) and level of trust in the government’s handling of the pandemic (lowest level versus highest [reference]: standardised score = − 0.29 [95% CI − 0.43, − 0.15]) (Additional file 1: Table 3).

## Discussion

In our survey, Norwegian baccalaureate nursing students reported significantly higher levels of fear of COVID-19 compared to urban Norwegian adults [27]. Our mean FCV-19S score of 2.45 is, however, lower than the mean score of 2.95, reported previously for Filipino nursing students [28]. Two similar studies, one in Spanish University students (12% nursing students) [41], and one in Russian/Belarussian young adults (28% university students) [42] reported levels of FCV-19S of 2.4 and 2.45, respectively, comparable to our findings in Norwegian nursing students.

Eight out of twelve background variables were significantly associated with fear of COVID‐19 in the baccalaureate nursing students. Fear of COVID-19 was more evident among first year nursing students, students under the age of 25 and students who did not trust the government’s or the universities’ handling of the COVID-19 situation. Fear of COVID-19 was also more prominent among students who reported feeling lonely due to COVID-19 and among those who had not been in clinical practice. Interestingly, higher health literacy was associated with lower FCV-19S scores in a cross sectional study of medical students in Vietnam [43].

Our findings are consistent with the results of several other studies of different psychological outcomes, such as anxiety, fear and stress in nursing students during the present pandemic in different countries [13, 17, 19, 20].

An important aspect is that the timely and strict national and regional measures have contributed to keeping the spread of COVID-19 low in Norway, as compared to other European countries [44]. Collectively, the results indicate that students who are young, lonely and less socially interactive than their peers are the most vulnerable individuals. A recently published systematic review of the effects of COVID-19 on psychological outcomes of the general population showed that the risk factors associated with distress measures included female gender, younger age group (≤ 40 years), presence of a chronic/psychiatric illness, student status and frequent exposure to social media/news concerning COVID-19 [45].

Baccalaureate nursing students had significantly worse scores on general health, psychological distress and overall QoL than did the gender- and age-adjusted reference data collected prior to the COVID-19 epidemic. However, the associations of the FCV-19S score with general health, psychological distress and overall QoL were small, moderate and trivial, respectively. The weak association between fear of COVID-19 and overall QoL indicates that other factors related to being a student during a pandemic may have larger effects. For example, a Polish study found that social distancing, self-isolation and limited access to public spaces among young university students were associated with decreased QoL [46]. Moreover, a study of nursing students in rural Appalachia, West Virginia, USA, suggested that factors such as resilience and preparedness for online learning were associated with QoL [47].

The pandemic caused significant disruptions in the daily lives of baccalaureate nursing students. First year students had to encounter a new reality just a few months after having established life as a student. All students were exposed to stressful factors, such as the closing of campuses and conversion of educational sessions to digital teaching. Some may have experienced cancellations of planned practical training, while others, by practical training, became part of the health care system where the pandemic led to an increased workload due to restrictions, frequent testing and an increased number of patients. In sum, such factors may have added to the students’ perceived fear. Fear is considered an adaptive normal response in the presence of danger or uncertainty but it can become burdensome if the threat is continuous and unpredictable [10], as in the current COVID-19 pandemic. Not knowing how long the pandemic will last, what consequences it may have for their personal health, progression in their studies and future working life probably raises students’ fear and concerns. However, knowledge and skills regarding infection control measures, a stable educational framework and continuing contact with the university staff through high quality distant teaching may support students during a challenging period [14].

The level of fear of COVID-19 between universities seemed to vary with the regional incidence of infection and level and duration of restrictions during the period in which the survey was conducted. Apart from their fear of Covid-19 scores, students in the capital area (attending Oslo Metropolitan University) also reported significantly higher levels of psychological distress (SCL-5) and worse overall QoL compared to those in the other universities (Fig. 1). This finding is consistent with studies of distress among Norwegian students in December 2020, revealing higher levels of psychological distress in the two largest cities where societal restrictions were most intrusive[17]. A study conducted in China among nurses and nursing students reported higher scores on measures of anxiety and anger in participants living in proximity to COVID-19 zones, i.e. areas with a higher prevalence of infection [13].

The emergence of the COVID-19 pandemic and its consequences can probably explain the absolute difference in scores between the baccalaureate nursing students and reference data. Most notably, QoL was reduced by 1.16 SDs (Table 2), which is interpreted as a large effect size. The level of psychological distress was significantly higher in our sample than in the pre-pandemic reference data reported by medical students [15], while general health was not as affected. The latter observation may be interpreted as general health being more of a physical measure, whereas fear in general, is more strongly associated with psychological distress. Our findings correspond to a recently published meta-analysis which reported an association of fear of COVID-19 with a wide range of mental health problems in the general population [5]. Experiences from previous pandemics, such as the MERS-CoV pandemic, indicate that higher education institutions (with health programmes) need to educate their students about effective crisis management and provide high quality and safe clinical learning environments [48]. Inadequate efforts to recognise and address college students’ mental health challenges, especially during a pandemic, could have long-term consequences on their health and education [48].

### Strengths and limitations of this study

This cross-sectional survey design is a limitation, as no changes over time, either from before or until the end of the pandemic, can be assessed. Nevertheless, the sample size (n = 2605), fairly high response rate and comparisons of students with reference data increases the reliability of our findings.

We used validated instruments for the subjective reporting of health and overall QoL, which support the quality of sufficient data. The data may help guide the balancing of infection control measures at higher education institutions during a pandemic, while at the same time protecting students’ needs. Large scale and longitudinal follow up studies are warranted.

## Study implications and conclusion

Research on baccalaureate nursing students’ subjective perceptions of health and overall QoL helped identify factors that may represent a threat to individual students. Self-reported data can have an important cognitive and practical value, and may contribute to the handling of ongoing and future pandemics [48]. To reduce fear and psychological distress, and to improve QoL among nursing students during a pandemic, closer follow-up of vulnerable students could be implemented by the universities. Our results indicate that special attention should be given to first year students, students reporting to feel lonely, students not engaged in clinical practice and those who report a low level of trust.

The Norwegian version of the FCV-19S [27] has been used to investigate the associations of fear of COVID-19 with self-reported health measures and QoL in nursing students. Compared to the reference data collected prior to the COVID-19 outbreak, our respondents reported significantly worse general health and overall QoL, and greater psychological distress. These differences were trivial for general health, moderate for psychological distress and large for QoL. The large difference in QoL between the nursing students who responded and the reference data was only slightly related to fear of COVID-19 scores, indicating that other factors, possibly related to being a student during a pandemic, might have contributed to the results. This possibility will be explored in future studies.

## Supplementary Information

**Additional file 1:Table 1.** Multiple hierarchical regression analysis with general health (z-score) as the dependent variable. **Table 2.** Multiple hierarchical regression analysis with psychological distress (SCL-5 z-score) as the dependent variable. **Table 3.** Multiple hierarchical regression analysis with overall quality of life (z-score) as the dependent variable.

## Data Availability

The dataset analysed during the current study are available from the corresponding author on reasonable request.
