# Supplementary material for: Quality of life and fear of COVID-19 in 2600 baccalaureate nursing students at five universities: a cross-sectional study
Source: Health Qual Life Outcomes. 2021 Aug 19;19:198. doi: 10.1186/s12955-021-01837-2 (PMC8374118; doi:10.1186/s12955-021-01837-2)
Supplement: Supplementary file 1 — Additional file 1:Table 1. Multiple hierarchical regression analysis with general health (z-score) as the dependent variable. Table 2. Multiple hierarchical regression analysis with psychological distress (SCL-5 z-score) as the dependent variable. Table 3. Multiple hierarchical regression analysis with overall quality of life (z-score) as the dependent variable. [file 12955_2021_1837_MOESM1_ESM.docx]

Supplements

Table 1. Multiple hierarchical regression analysis with general health (z-score)

as the dependent variable

| Variables | Standardized coefficient. (95% CI) | Overall P-value |
| --- | --- | --- |
| FCV-19S (continuous z-score) | -0.13 (-0.17, -0.09) | <0.001 |
| Years in nursing school    1    2    3 | Ref.  0.05 (-0.07, 0.18)  0.05 (-0.08, 0.17) | 0.680 |
| Age, years    <25    25-29    ≥30 | Ref.  -0.05 (-0.16, 0.05)  -0.17 (-0.28, -0.06) | 0.007 |
| Living alone    No    Yes | Ref.  -0.01 (-0.10, 0.09) | 0.903 |
| Number of times tested for COVID-19    Never    1    2    3    ≥ 4 | Ref.   -0.10 (-0.19, 0.00)  -0.06 (-0.14, 0.06)  -0.06 (-0.19, 0.07)  -0.07 (-0.20, 0.06) | 0.448 |
| History of positive Covid-19 test    No    Yes | Ref.  -0.04 (-0.21, 0.13) | 0.669 |
| Quarantine status related to COVID-19    Never    Previous    Present | Ref.  0.05 (-0.03, 0.12)  0.05 (-0.22, 0.32) | 0.552 |
| At risk of COVID-19 complications    No    Uncertain    Yes | Ref.  --0.51 (-0.62, -0.40)  -0.77 (-0.91, -0.63) | <0.001 |
| Trust in governmental handling the COVID-19 situation    Strongly disagree/disagree    Neither disagree nor agree    Agree    Strongly agree | Ref.  -0.05 (-0.19, 0.09)  0.13 (-0.01, 0.26)  0.37 (0.22, 0.52) | <0.001 |
| Trust in universities’ handling of the COVID-19 situation    Strongly disagree    Disagree    Neither disagree nor degree    Agree    Strongly agree | Ref.  -0.02 (-0.18, 0.14)  0.03 (-0.12, 0.18)  0.09 (-0.06, 0.24)  0.17 (-0.02, 0.35) | 0.097 |
| Feeling lonely due to COVID-19    Strongly disagree    Disagree    Neither disagree nor degree    Agree    Strongly agree | Ref.  -0.21 (-0.38, -0.05)  -0.28 (-0.44, -0.11)  -0.35 (-0.51, -0.20)  -0.47 (-0.64, -0.31) | <0.001 |
| Engagement in clinical practice during the pandemic    Yes    No | Ref.  0.07 (-0.05, 0.18) | 0.256 |

Table 2. Multiple hierarchical regression analysis with psychological distress (SCL-5 z-score) as the dependent variable

| Variables | Standardized coefficient. (95% CI) | Overall P-value |
| --- | --- | --- |
| FCV-19S (continuous z-score) | 0.38 (0.34, 0.41) | <0.001 |
| Years in nursing school    1    2    3 | Ref.  -0.09 (-0.19, 0.02)  -0.10 (-0.20, 0.01) | 0.182 |
| Age, years    <25    25-29    ≥30 | Ref.  -0.06 (-0.14, -0.03)  -0.24 (-0.33, -0.15) | <0.001 |
| Living alone    No    Yes | Ref.   -0.08 (-0.15, 0.00) | 0.060 |
| Number of times tested for COVID-19    Never    1    2    3    ≥ 4 | Ref.   0.04 (-0.04, 0,12)  0.02 (-0.08, 0.11)  0.03 -0.07, 0.14)  0.07 (-0.03, 0.18) | 0.709 |
| History of positive Covid-19 test    No    Yes | Ref.  -0.05 (-0.19, 0.10) | 0.523 |
| Quarantine status related to COVID-19    Never    Previous    Present | Ref.  -0.02 (-0.08, 0.05)  0.19 (-0.04, 0.41) | 0.198 |
| At risk of COVID-19 complications    No    Uncertain    Yes | Ref.  -0.02 (-0.11, 0.07)  0.05 (-0.06, 0.17) | 0.563 |
| Trust in governmental handling the COVID-19 situation    Strongly disagree/disagree    Neither disagree nor agree    Agree    Strongly agree | Ref.  -0.12 (-0.24, -0.00)  -0.21 (-0.21, -0.10)  -0.28 (-0.41, -0.15) | <0.001 |
| Trust in universities’ handling of the COVID-19 situation    Strongly disagree    Disagree    Neither disagree nor degree    Agree    Strongly agree | Ref.  -0.05 (-0.18, 0.09)  -0.08 (-0.21, 0.05)  -0.21 (-0.33, -0.08)  -0.17 (-0.32, -0.01) | <0.001 |
| Feeling lonely due to COVID-19    Strongly disagree    Disagree    Neither disagree nor agree    Agree    Strongly agree | Ref.  0.16 (0.02, 0.31)  0.28 (0.14, 0.42)  0.56 (0.43, 0.69)  1.01 (0.88, 1,.15) | <0.001 |
| Engagement in clinical practice during the pandemic    Yes    No | Ref.  0.05 (-0.05, 0.15) | 0.296 |

Table 3. Multiple hierarchical regression analysis with overall quality of life (z-score) as the dependent variable

| Variables | Standardized coefficient (95% CI) | Overall P-value |
| --- | --- | --- |
| FCV-19S (continuous z-score) | -0.09 (--0.13, -0.06) | <0.001 |
| Years in nursing school    1    2    3 | Ref.  0.02 (-0.9, 0.13)  0.08 (-0.3, 0.19) | 0.235 |
| Age, years    <25    25-29    ≥30 | Ref.  0.06 (-0.03, 0.16)  0.15 (0.05, 0.24) | 0.009 |
| Living alone    No    Yes | Ref.  -0.03 (-0.11, 0.06) | 0.515 |
| Number of times tested for COVID-19    Never    1    2    3    ≥ 4 | Ref.  (-0.09, 0.09)  0.03 (-0.07, 0.13)  0.00 (-0.11, 0.12)  -0.07 (-0.18, 0.04) | 0.573 |
| History of positive Covid-19 test    No    Yes | Ref.  0.03 (-0.13, 0.18) | 0.744 |
| Quarantine status related to COVID-19    Never    Previous    Present | Ref.  -0.09 (-0.33, 0.15)  -0.07 (-0.31, 0.17) | 0.700 |
| At risk of COVID-19 complications    No    Uncertain    Yes | Ref.  -0.12 (-0.22, -0.02)  -0.11 (-0.24, 0.02) | 0.022 |
| Trust in governmental handling the COVID-19 situation    Strongly disagree/disagree    Neither disagree nor agree    Agree    Strongly agree | Ref.  0.07 (-0.62, 0.19)  0.21 (0.09, 0.33)  0.29 (0.15, 0.43) | <0.001 |
| Trust in universities’ handling of the COVID-19 situation    Strongly disagree    Disagree    Neither disagree nor degree    Agree    Strongly agree | Ref.  0.03 (-0.11, 0.17)  0.03 (-0.10, 0.17)  0.24 (0.10, 0.38)  0.20 (0.03, 0.37) | <0.001 |
| Feeling lonely due to COVID-19    Strongly disagree    Disagree    Neither disagree nor degree    Agree    Strongly agree | Ref.  -0.29 (-0.45, -0.14)  -0.46 (-0.61, -0.31)  -0.82 (-0.96, -0.68)  -1. 38 (-1.52, -1.23) | <0.001 |
| Engagement in clinical practice during the pandemic    Yes    No | Ref.  0.05 (-0.06, 0.15) | 0.377 |
